# Notch intracellular domains form transcriptionally active heterodimeric complexes on sequence-paired sites

**DOI:** 10.1038/s41598-023-50763-4

**Published:** 2024-01-02

**Authors:** Tana R. Gazdik, Jacob J. Crow, Tyler Lawton, Chloe J. Munroe, Hannah Theriault, Travis M. Wood, Allan R. Albig

**Affiliations:** 1 Biomolecular Sciences PhD Program, Boise State University, Boise, ID 83725 USA; 2 Department of Biological Sciences, Boise State University, Boise, ID 83725 USA; 3 Department of Chemistry, Boise State University, Boise, ID 83725 USA

**Keywords:** Cell signalling, Transcription

## Abstract

Notch signaling is universally conserved in metazoans where it is important for a wide variety of both normal and abnormal physiology. All four mammalian Notch receptors are activated by a conserved mechanism that releases Notch intracellular domains (NICDs) from the plasma membrane to translocate to the nucleus. Once there, NICDs interact through highly conserved ankyrin domains to form head-to-head homodimers on Notch sensitive promoters and stimulate transcription. Due to the highly conserved nature of these Notch ankyrin domains in all four mammalian Notch proteins, we hypothesized that NICDs may also engage in heterodimerization. Our results reveal the presence of two NICD dimerization states that can both engage in homo and heterodimerization. Using a Co-IP approach, we show that all NICD’s can form non-transcriptionally active dimers and that the N4ICD appears to perform this function better than the other NICDs. Using a combination of ChIP analysis and transcriptional reporter assays, we also demonstrate the formation of transcriptionally active heterodimers that form on DNA. In particular, we demonstrate heterodimerization between the N2ICD and N4ICD and show that this heterodimer pair appears to exhibit differential activity on various Notch sensitive promoters. These results illustrate a new diversification of Notch signaling mechanisms which will help us better understand basic Notch function.

## Introduction

The Notch signal transduction system is an ancient cell–cell communication mechanism that is conserved in essentially all metazoans^1,2^. Under normal conditions, Notch serves a wide variety of roles in development, vascular biology, and immune function^3^. In contrast, dysregulation of Notch is linked to a variety of diseases^3^. A clearer understanding of Notch signaling will provide new insights into the diverse processes governed by this pathway.

There are four individual Notch receptors (Notch1-4) present in mammalian genomes. Notch1 and Notch2 are highly similar molecules, while Notch3 and Notch4 are more divergent^4^. All Notch receptors are thought to be activated by the same mechanism involving juxtracrine interactions between adjacent cells when a Notch ligand on one cell is presented to a Notch receptor on a neighboring cell. Following ligand-receptor interaction, endocytosis of the Notch ligand provides ~ 4–12 pN of force^5^ which leads to the unfolding of the negative regulatory region of the receptor, thereby exposing the S2 and S3 cleavage sites. (reviewed in^6^). The solubilized cytoplasmic S3 fragment (Notch intracellular domain, NICD) then translocates to the nucleus and participates in a tripartite co-transcriptional complex with the DNA binding protein RBPJ and the transcriptional activator MAML^7,8^. This complex interacts with an 8 bp-optimized sequence (*i.e.,* TP-1 elements) on the DNA^9^, that can be found as monomers, or as dimers arranged in a head-to-head orientation (known as SPS sequence-paired sites) that generate a synergistic activation of Notch responsive promoters.

Experimental evidence from EMSA and crystallography studies have shown that NICD tripartite complexes can form head-to-head homodimers on SPS sites and that head-to-head dimerization is important for strong transcriptional activation of SPS sites^10–14^. NICD dimerization occurs between the ankyrin domains of interacting NICD molecules and both EMSA and crystallographic studies have confirmed the importance of ankyrin domains in NICD dimerization and transcription from sequence paired sites^12,13^. The broader significance of NICD dimerization through ankyrin interactions is illustrated by the observations of Liu et al.^15^, who found that NICD dimerization is required for Notch induced leukemogenesis and T-cell development. Sequences in the more divergent N- and C-terminals of Notch receptors have also been implicated in NICD homo-dimerization although this is less well understood^16^. Given the high degree of conservation within the ankyrin domains of all four mammalian Notch receptors, a long-outstanding question is whether different NICD molecules can engage in homodimeric as well as heterodimeric complexes. This question is especially important since the four mammalian Notch proteins are known to have transcriptional inequalities thus raising the possibility that heterodimeric NICD interactions may serve to diversity the Notch signaling mechanism.

The goal of this investigation was to test the hypothesis that the NICD molecules can engage in heterodimeric interactions. Using a combination of immunoprecipitation, chromatin IP, and luciferase reporter assays, we found that the NICDs are capable of interacting in both homo- and heterodimeric complexes. The implications of this result are currently unknown, however, given that we and others show that many cells express more than one type of Notch receptor and that all four Notch proteins have variable transcriptional activities^17^, it is possible that heterodimerization of NICDs may serve to diversify the overall Notch signaling mechanism.

## Materials and methods

### Cell culture

HEK293T cells were cultured in Dulbecco’s Modified Eagle’s medium (DMEM) supplemented with 10% EqualFETAL (Atlas Biologicals) and 1 × penicillin–streptomycin solution. Cells were grown in 10 cm plates and passaged at 80–90% confluence.

### Plasmids and plasmid mutagenesis

The FLAG-NICD constructs were all gifts from Raphael Kopan^10^ and acquired from Addgene. N1ICD (Addgene #20183), N2ICD (#20184), N3ICD (#20185), and N4ICD (#20186) all have an N-terminal 3×FLAG tag and code for the transcriptionally active, intracellular domain of the mouse Notch proteins. The NICD coding regions were then subcloned into pKH3 (#12555), a gift from Ian Macara^18^ to add a C-terminal 3×HA tag as well as into pcDNA3.1/MYC-His (Invitrogen) to add a C-terminal MYC-His tag. The C-terminally truncated N1ICD-MYC construct was given to us by Raphael Kopan^19^ (#41730) and encodes for the mouse N1ICD from V1744 to S2184.

The mouse Notch ankyrin domain mutants were created by aligning the human and mouse N1ICD to find the equivalent residues involved in N1ICD dimerization^12,20^. Afterwards, the mouse NICDs were aligned to find the equivalent residues across the other mouse NICDs. N1ICD (R1974A), N2ICD (R1934A), N3ICD (R1896A), and N4ICD (R1685A) mutants were all created through site-directed mutagenesis in each tagged-variation of the NICD (FLAG/HA/MYC).

The mouse Notch homodimerization mutants were created using the same method as above, where the mouse NICDs were aligned to the human N1ICD to find the conserved amino acids crucial to NICD interaction. The mouse NICD mutants are N1ICD (E1939K), N2ICD (K1895E, D1899K), N3ICD (K1857E, D1861K), and N4ICD (R1646E, E1650K). These mutants were created from both the FLAG-tagged and MYC-tagged variations of NICD.

For the transcriptional reporter assays, Hes5-Luc (Addgene #41724) and Hes1-Luc (Addgene #41723) were gifts from Ryoichiro Kageyama and Raphael Kopan. These luciferase reporters contain portions of the native mouse Hes5/Hes1 promoter and include several RBPJ binding sites to report the activity of Notch signaling. The Hes4-Luc reporter (− 139 to − 9) was cloned by PCR amplification of the ChIP-isolated Hes4 DNA shown in Fig. 8. This PCR fragment was cloned into pGL3-basic (Promega) with 5’Kpn1 and 3’SacI sites. The SPS-Core (16 and 21 basepair gaps) reporters were constructed by oligo hybridization and standard cloning techniques as previously described^17^. The CMV-β-gal plasmid was originally from Clontech.

All primers used for vector cloning and NICD mutagenesis can be found in Supplementary Tables 3–5.

### RT-PCR analysis

A variety of cell lines were grown in order to collect their mRNA and analyze their Notch expression profiles. Cells were plated in 10 cm dishes and grown until 80% confluence. To collect the cells and their mRNA, they were washed with 1×PBS and lysed with RiboZol RNA Extraction Reagent (Amresco). mRNA was collected and purified following the manufacturer’s recommended protocol. Equivalent amounts of RNA were reverse transcribed with iScript (Bio-Rad) following the manufacturer’s protocol and diluted for PCR analysis. Primers used to target human Notch mRNA are listed in Supplementary Table 1. Primers targeting 18S rRNA was used as an internal control to check cDNA quality.

### Immunoprecipitations

Cells were plated into 6-well plates at a density of 300,000 cells/well. The following day, cells were transfected with polyethylenimine (PEI, Polysciences) at a ratio of 5 μg PEI for every 1 μg plasmid DNA. NICD expression constructs were transfected at varying amounts in an attempt to equalize cellular protein expression based on preliminary titration/western blot experiments as follows (750 ng N1ICD, 500 ng N2ICD, 500 ng N3ICD, 250 ng N4ICD). Forty-eight hours after transfection, cell lysates were prepared for immunoprecipitations and performed as previously described^21^.

### Western blotting

Prepared protein lysates and Co-IP samples were separated through SDS PAGE gels. Samples were then blotted onto nitrocellulose membranes and blocked in TBS-T (140 mM NaCl, 25 mM Tris–HCl, pH 7.4, 0.1% Tween-20) with 5% bovine serum albumin. Membranes were incubated with primary antibody (1:500; MYC, 1:1000; FLAG, 1:2000; HA) overnight on a rotator at 4 °C. Following incubation, membranes were washed 3 × 10 min in TBS-T and then incubated with a secondary antibody solution, composed of horseradish peroxidase conjugated secondary antibodies at a concentration of 1:10,000. Membranes were then washed again 3 × 10 min in TBS-T and proteins were detected through enhanced chemiluminescence.

### Antibodies

For western blotting, primary antibodies against FLAG(DYKDDDDK)-tag (D6W5B) were purchased from Cell Signaling Technology, while antibodies against HA(YPYDVPDYA)-tag (Y-11, sc-805) and MYC(EQKLISEEDL)-tag (9E10, sc-40) were purchased from Santa Cruz Biotechnology. Secondary antibodies were purchased from GE Healthcare Life Sciences and consisted of α-mouse (NA931V) or α-rabbit (NA934V) horseradish peroxidase conjugated antibodies. The anti-FLAG antibody resin used for CoIP was from Genscript.

### Chromatin IP

Chromatin immunoprecipitation (ChIP) analyses were performed as previously described^22^, with minor modifications including omission of SDS from the dilution, high-salt wash, and elution buffers, and omission of spermidine from the micrococcal nuclear buffer. 293T cells were transfected with two differentially tagged NICDs and then 48 h later were fixed by formaldehyde-induced crosslinking and the nuclear fraction was isolated and collected. Micrococcal nuclease (NEB) was used to cut the chromatin into 300–1500 base pair fragments, the nucleus briefly sonicated to lyse the fraction, and the DNA collected. To specifically isolate the dimerized complexes bound to DNA, they were placed through two rounds of selection, targeting both partners within the Notch dimer. The first round of selection was performed with anti-FLAG G1 affinity resin (GenScript), samples thoroughly washed, and complexes eluted with 3×FLAG-peptide (ApexBio). Those complexes were placed through a second round of selection, probing for a HA-tagged NICD partner, with biotinylated HA antibodies (Bioss). These targets were precipitated out of solution with streptavidin magnetic beads (NEB), thoroughly washed again, and the DNA was isolated out of the complexes with Proteinase K (Amresco) and reverse crosslinking. DNA was cleaned and purified with a PCR purification kit and samples were analyzed for Notch-dimer targets using Hes1 or Hes4 primers as indicated in supplementary table 2.

### Luciferase assays

Cells were plated in 24-well plates at 50,000 cells/well. The following day, cells were transfected with PEI at a ratio of 5 μg PEI for every 1 μg DNA. Cells were transfected with 100 ng/well luciferase promoter and 50 ng/well of the various NICD constructs. Wells that had two constructs were transfected with 25 ng/well of each construct. In order to normalize data for transfection efficiency and potential cell growth/death, we co-transfected with 10 ng/well of a CMV-β-Galactosidase construct. Cells were lysed 48 h post transfection in 1× firefly luciferase lysis buffer (Biotium). Lysates were analyzed following the manufacturer’s protocol (Firefly Luciferase Assay Kit, Biotium) using a Promega© Glomax Multi Detection System luminometer. Luciferase activity was normalized to β-Galactosidase activity (Promega) and values were reported as a fold change to control. In order to minimize transfection variations and output noise, all conditions were performed in triplicate for each independent experiment.

## Results

### Cellular expression of multiple Notch isoforms

Previous work revealed the now canonical crystal structure of the mammalian Notch transcriptional complex containing fragments of RBPj, MAML1, and the N1ICD ankyrin domains in a head-to-head dimerized state^12,13^. Nam et al.^12^, also defined four key residues in the N1ICD ankyrin domain critical for dimerization of N1ICD molecules and determined that these residues are highly conserved in the NICD ankyrin domains of all four mammalian Notch isoforms. This data suggested that all Notch NICD domains likely engage in head-to-head dimerization interactions similar to N1ICD. Furthermore, given the overall sequence similarity of the Notch ankyrin domains and the conservation of these key amino acids required for dimerization (Supplementary Fig. 1), it was further hypothesized that NICD domains may also be able to engage in heterodimeric interactions, although this has not been investigated.

**Figure 1:**
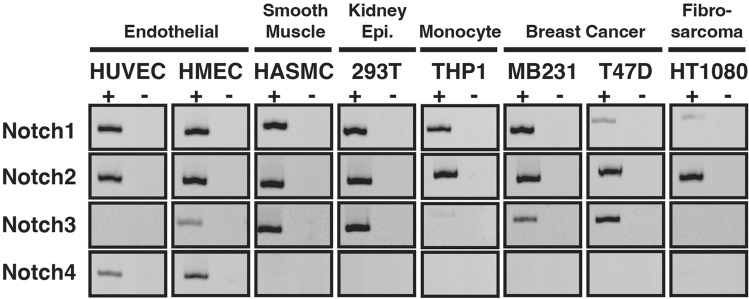
Multiple cell lines express more than one Notch isoform. RT-PCR was used to monitor expression of Notch proteins in cell lines from various tissues. + /− denotes + RT experimental samples and -RT control samples. Image shown represents data from two independent repetitions.

In order for heterodimeric NICD interactions to be even feasible, at least two isoforms of Notch receptors would need to be expressed in a single cell. To assess the expression of Notch receptors in single cell lines, and thus the possibility of NICD heterodimerization, we screened several cell lines for their potential for co-expression of Notch receptors. Reverse-Transcription PCR (RT-PCR) was used to monitor mRNA expression of each Notch protein. As shown in Fig. 1, each of the cell lines examined expressed mRNA for at least two Notch receptors, while several expressed three or even all four Notch receptors. These results suggested that cellular expression of multiple Notch isoforms is common and solidified the possibility that heterodimeric interactions between different NICDs is at least feasible.

### Co-immunoprecipitation of NICD heterodimer complexes

**Figure 2:**
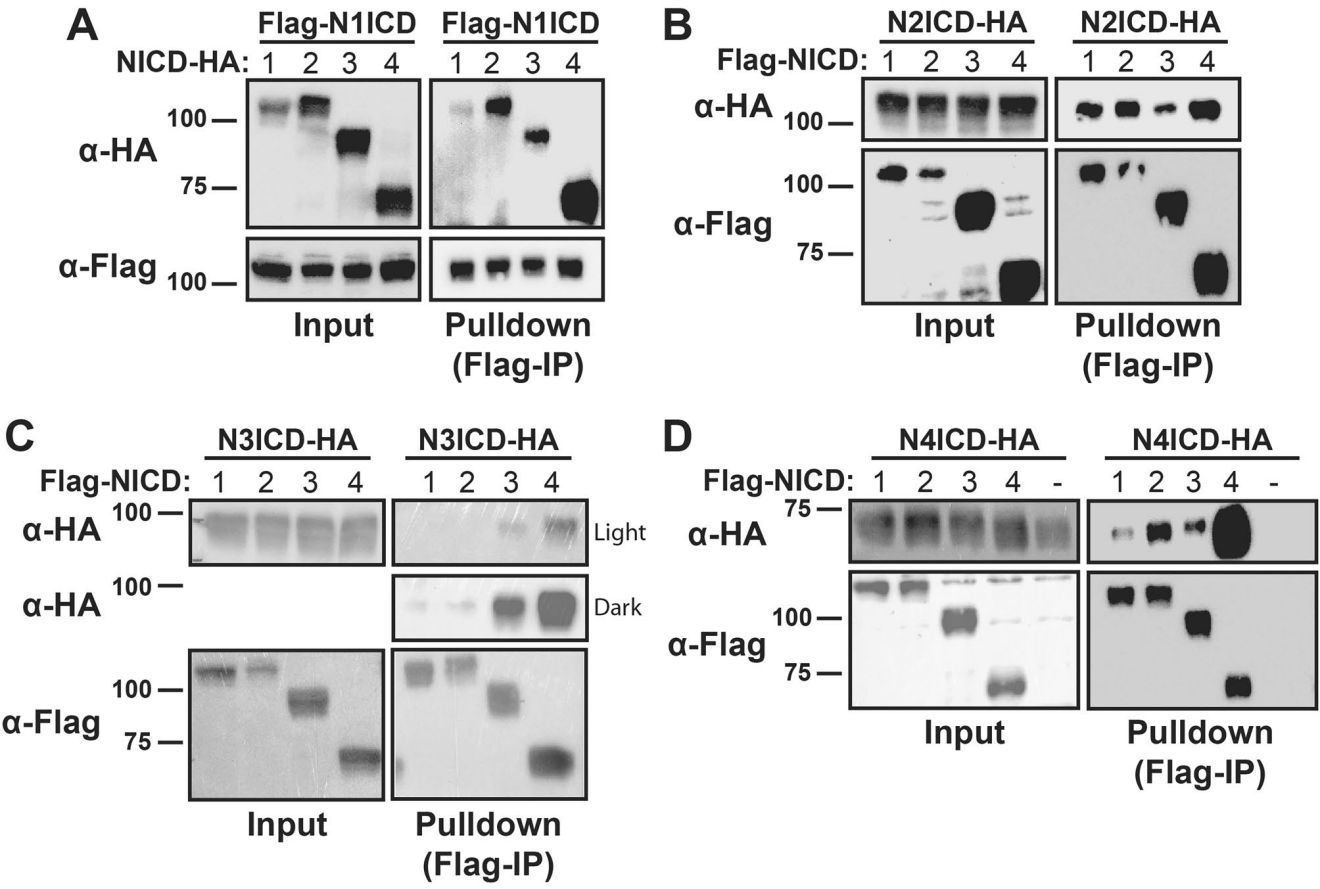
Detection of NICD homo- and heterodimer complexes. 293T cells were transfected with FLAG- and HA- tagged species of various NICDs and dimerization was detected by co-immunoprecipitation with anti-FLAG antibodies and western blotting with anti-HA antibodies. In all panels, Input represents 10% of whole cell lysates before Co-IP. The heterodimerization possibilities are represented with (**A**) N1ICD, (**B**) N2ICD, (**C**) N3ICD, and (**D**) N4ICD. To prevent confounding results from residual unstripped antibodies, lysates were run twice on two different membranes for cleaner western blot analysis. Panel D also includes an example of a typical negative FLAG-tagged NICD lane (-), controlling for non-specific interactions between HA-tagged protein on anti-FLAG antibody resins.

To test the hypothesis that the NICD domains can engage in heterodimeric interactions, we first established a cellular co-immunoprecipitation (Co-IP) strategy that involved the generation of FLAG, MYC, and HA-tagged versions of each of the four different NICD molecules. Co-transfection of 293T cells with two different tags would thus allow us to simultaneously monitor NICD homodimerization and heterodimerization in the precipitates. 293T cells were transiently co-transfected with cDNAs encoding one FLAG-tagged and a second MYC- or HA-tagged pair of NICD proteins. Anti-FLAG Co-IP was used to isolate FLAG-tagged NICDs, then immunoblotting with anti-MYC or anti-HA antibodies was used to detect MYC- or HA-tagged Co-IP NICD partners, depending on combination. As shown in Fig. 2A, full-length FLAG-N1ICD engaged in homodimeric interactions with full-length HA-tagged N1ICD and heterodimeric interactions with the HA-tagged N2, N3, and N4ICD molecules. Interestingly, N1ICD appeared to co-immunoprecipitate with N2, N3, and N4ICD better than with N1ICD itself suggesting that N1ICD may preferentially engage in heterodimeric interactions. Moreover, N1ICD appeared to most strongly precipitate with N4ICD. N2ICD also formed homodimers and heterodimers with each of the other NICDs and again, the N2ICD-N4ICD interaction appeared to be the most robust (Fig. 2B). N3ICD formed heterodimers with each of the other NICDs (Fig. 2C) although dimerization with N1ICD and N2ICD appeared to be weak (Fig. 2C dark) and as before, the interaction between N3ICD and N4ICD appeared to be the strongest (Fig. 2C light). Finally, N4ICD demonstrated strong heterodimerization with the other NICDs, but again, N4ICD homodimerization appeared to be the most robust (Fig. 2D). Importantly, Co-IP of MYC and HA-tagged NICDs was consistently dependent on the presence of FLAG co-transfection thus illustrating the specificity of the co-immunoprecipitation system.

Collectively, this series of Co-IP experiments demonstrated that all NICDs appear to be capable of mix and match homodimer and heterodimer interactions. Moreover, these results also showed that the NICDs do not appear to equally engage in dimerization and that N4ICD appeared to consistently display the most robust homodimerization and heterodimerization compared to the other NICD molecules. Given the variability of NICD expression associated with transient transfection however, the relative strength of NICD dimer interactions will need to be validated under more precise conditions.

### Ankyrin domains are not required for NICD Co-IP

Although the canonical and transcriptionally active NICD tripartite complex is the most studied Notch dimer complex, another less well studied dimer complex was previously described by Vasquez et al.^16^, This alternative NICD dimer complex was identified by co-immunoprecipitation methods similar to those used above and was described as an “antiparallel” complex since it involved the dimerization of two N1ICD monomers via N-terminal to C-terminal interactions such that the dimerizing molecules were proposed to orientate in an antiparallel configuration reminiscent of a “yin-yang” symbol. In this yin-yang conformation, the ankyrin domains which mediate the tripartite complex head-to-head NICD interactions were found to be non-essential for dimerization^16^.

**Figure 3:**
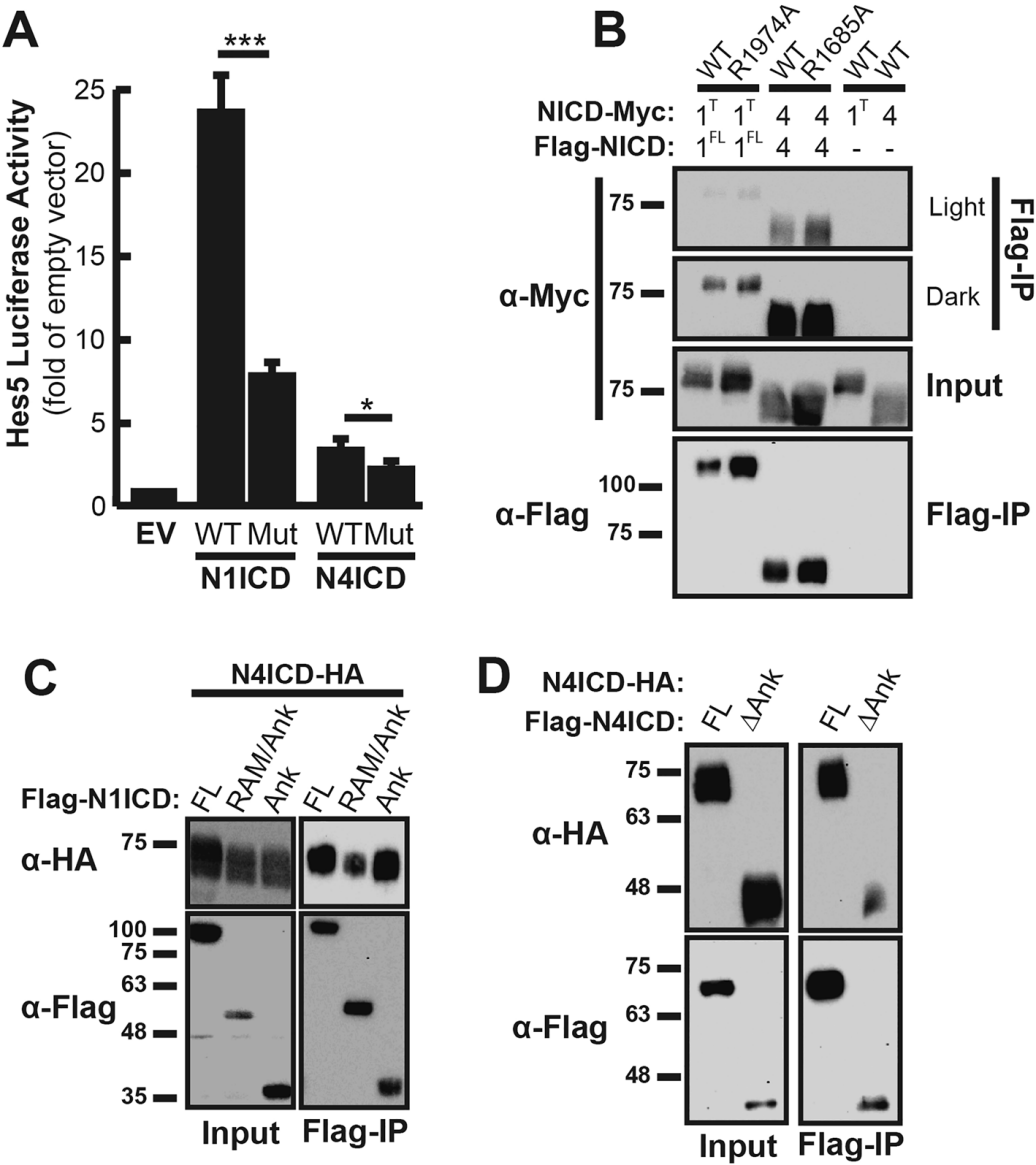
Ankyrin domains are not required for NICD co-immunoprecipitation. (**A**) 293T cells were co-transfected with Hes5-promoter luciferase reporter and wild-type (WT) or corresponding ankyrin domain mutant (Mut) NICDs. Reporter activation is compared to its basal activity in cells, which received an equivalent amount of empty overexpression vector (EV). Shown are the average + /− SE of six experiments. Transfection efficiency was normalized by co-transfection with CMV-β-gal reporter plasmids and measuring β-gal activity. Statistical significance was determined through a student’s two-tailed t test, where *** is p < 0.001, and * is p < 0.05. (**B**) 293T cells were transfected with pairs of cDNAs encoding FLAG- and MYC-tagged WT NICD domains or mutant FLAG- and MYC-tagged mutant ankyrin NICD domains. Anti-FLAG antibodies were used to isolate FLAG-tagged proteins and western blotting with anti-MYC antibodies was used to detect interacting NICD domains. Shown is a representative image of a single experiment that was performed four independent times. To account for dimerization intensities, the figure includes a light and dark exposure of the co-immunoprecipitated MYC-partners. (**C**) 293T cells were transfected with HA-tagged N4ICD and various FLAG-tagged N1ICD constructs including the full-length (FL) N1ICD, N1ICD lacking the C-terminus (RAM/Ank), and the isolated N1ICD ankyrin domain (Ank). Anti-FLAG antibodies were used to detect interacting HA-tagged molecules. Shown is a representative image of a single experiment that was performed three times. (**D**) 293T cells were transfected with full-length HA-tagged N4ICD and either full-length or ankyrin deletion mutant (ΔAnk) FLAG-tagged N4ICD. Co-IP and western analysis were performed as above. In all panels, Input represents 10% of whole cell lysate from each sample as a control for protein expression.

Based on these findings, it was important to determine if our co-immunoprecipitation experiments were detecting NICD dimers in the transcriptionally active head-to-head conformation, the yin-yang conformation, or perhaps a blend of both dimerization modalities. To accomplish this, we again performed the co-immunoprecipitation experiment with NICD molecules containing ankyrin domain mutations that are unable to form head-to-head dimer complexes. Arg1985 within the human N1ICD ankyrin domain was previously identified as important for NICD dimerization and transcriptional responses from promoters with paired RBPJ binding sites such as the Hes5 promoter^12,13^. Using sequence alignments, we found equivalent arginine residues in the mouse Notch1 and Notch4 NICDs (Arg1974 and Arg1685 respectively) (Supplementary Fig. 1) and performed site-directed mutagenesis to change these arginine residues to alanine residues. To confirm that these mutations decreased transcriptional activity from paired RBPJ sites and therefore head-to-head dimerization, we used a luciferase assay to monitor transcriptional activity from the SPS-containing Hes5 promoter. As shown in Fig. 3A, ankyrin mutant N1ICD demonstrated much weaker transcriptional activity compared to wild-type N1ICD. Moreover, ankyrin mutant N4ICD was also less efficient at driving Hes5 promoter activity, although the inherently low transcriptional activity of wild-type N4ICD made this effect less obvious compared to N1ICD. Since even non-dimerizing NICD domains minimally promote transcription, we were not surprised that these mutations failed to eliminate transcriptional activity altogether. Nonetheless, these results confirm that mutation of N1ICD and N4ICD at these positions decreases transcriptional activity by disrupting head-to-head dimerization of NICDs.

We next sought to determine if ankyrin domains are required for NICD co-immunoprecipitation. To accomplish this, we again performed the Co-IP assay to compare the ability of wild-type and ankyrin mutant N1ICD and N4ICD to Co-IP in 293T cells (Fig. 3B). 293T cells were transiently co-transfected with cDNAs encoding one FLAG-tagged and a second MYC- or HA-tagged pair of NICD molecules. Anti-FLAG Co-IP was again used to isolate FLAG-tagged NICDs, then immunoblotting with anti-MYC or anti-HA antibodies was used to detect MYC- or HA-tagged Co-IP NICD partners, depending on combination. Due to inconsistencies in observing full-length N1ICD homodimers on Western Blots, which we will discuss later, we found that a C-terminally truncated N1ICD construct lacking its PEST domain more reliably precipitated was employed for this experiment. Despite the importance of ankyrin domains for transcriptional activity and dimerization, we were unable to detect any change in N1ICD or N4ICD homodimerization when the ankyrin domains were mutated. This result suggested that ankyrin domains are not required for co-immunoprecipitation but did not rule out the possibility that ankyrin domains may at least participate in Co-IP. To determine if ankyrin domains are capable of mediating Co-IP, we therefore compared the ability of HA-tagged N4ICD to Co-IP with various isolated fragments of the N1ICD. Consistent with the results of Vasquez et al.^16^, we found that full-length N4ICD was able to interact with an N-terminal fragment of N1ICD containing just the RAM/Ank domains as well as with the isolated N1ICD ankyrin domain itself (Fig. 3C). Furthermore, as shown in Fig. 3D, complete ablation of the N4ICD ankyrin domain reduced, but did not abolish Co-IP between N4ICD molecules. Taken together, these results suggested that NICD ankyrin domains are not required for Co-IP between NICD complexes but are capable of mediating Co-IP and therefore, that our Co-IP procedure was likely recovering both the head-to-head and yin-yang dimer complexes.

### NICD molecules heterodimerize on DNA

**Figure 4:**
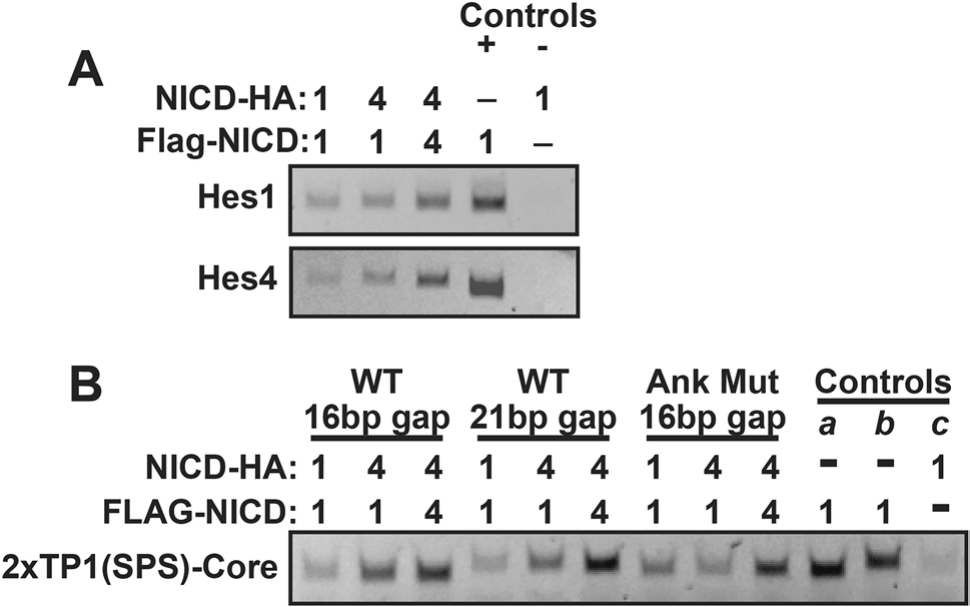
Detection of NICD heterodimer complexes on DNA. (**A**) 293T cells were transfected with combinations of FLAG-tagged N1ICD or N4ICD and FLAG- or HA-tagged binding NICD partners. ChIP was performed by two-step Co-IP with anti-FLAG then anti-HA antibodies. A positive control reaction was transfected with FLAG-tagged N1ICD alone and subjected to anti-FLAG Co-IP. A negative control reaction was transfected with HA-tagged N1ICD and subjected to anti-FLAG and anti-HA Co-IP. Hes1 and Hes4 promoter sequences were detected by PCR with antibodies flanking the head-to-head binding sites in each promoter. Shown is a representative image of a single experiment that was performed three independent times with similar results (**B**) 293T cells were transfected with combinations of FLAG-tagged N1ICD or N4ICD and FLAG- or HA-tagged binding NICD partners. ChIP was performed by two-step Co-IP with anti-FLAG then anti-HA antibodies. Positive control reactions (a and b) were transfected with FLAG-tagged N1ICD alone and subjected to anti-FLAG Co-IP. A negative control reaction (c) was transfected with HA-tagged N1ICD and subjected to anti-FLAG and anti-HA Co-IP. SPS 16 and SPS 21 promoter sequences were detected by PCR with antibodies flanking the head-to-head binding sites in each promoter. Shown is a representative image of a single experiment that was performed three independent times with similar results.

Our results indicated that dimerization between NICD molecules (as measured by Co-IP) did not directly correlate to transcriptional activity, that ankyrin domains were not required for Co-IP, and yet that isolated ankyrin domains could dimerize. Taken as a whole, these results suggested that NICDs may simultaneously exist in multiple dimerization states. One state was detected by co-immunoprecipitation but did not apparently correspond to transcriptional activity (yin-yang dimers) while the second state appeared to be transcriptionally active and dependent on interactions between ankyrin domains (head-to-head dimers). These observations raised the important question whether or not any of the NICD complexes that were being recovered by Co-IP were engaged in heterodimer complexes on DNA or rather were strictly in a non-transcriptional yin-yang complex as suggested by Vasquez et al.^16^. To address this question, we adapted our Co-IP procedure into a chromatin IP experiment to determine if any co-immunoprecipitating NICDs were bound to DNA. 293T cells were co-transfected with combinations of FLAG-tagged N1ICD or N4ICD coupled with HA-tagged N1ICD or N4ICD and simultaneously with a plasmid containing either the Hes4 or Hes1 SPS promoters. After crosslinking and DNA fragmentation, cell lysates were immunoprecipitated first with anti-FLAG antibodies then with anti-HA antibodies such that DNA would only be recovered if it was associated with both FLAG and HA-tagged NICD proteins. Control samples included transfection with FLAG-tagged N1ICD alone and a single anti-FLAG immunoprecipitation step (positive control) and a negative control that was transfected with HA-tagged NICD and subjected to the two-step Co-IP procedure. In all cases, precipitated DNA was detected by PCR with oligos that were designed to flank the paired head-to-head SPS binding sites within the Hes4 and Hes1 promoters. As shown in Fig. 4A, both Hes1 and Hes4 sequences were detected in the precipitated chromatin of all NICD combinations and the ChIP was specific since Hes4 and Hes1 promoters were not recovered if a FLAG-tagged protein was omitted.

On the surface, this result seemed to confirm that NICD molecules engage in homodimer complexes but also supported the hypothesis that NICD heterodimers may form on DNA. Despite this, it was important to investigate whether the captured NICD molecules were actually engaged in dimerized complexes or instead were individually binding to DNA in a non-dimerized state. To investigate this possibility, we performed another Co-IP experiment comparing NICD binding to SPS promoters with 16 or 21 bp (basepair) gaps separating the RBPj binding sites. 16 bp gaps have been shown to be optimal for supporting dimerization and transcriptional activity whereas 21 bp gaps are too wide to support dimerization and only very weakly promote transcription^17^. In addition, we also compared wild-type NICD proteins to their transcriptionally inactive ankyrin mutant versions. As shown in Fig. 4B, NICD homodimer and heterodimer complexes were detected on both the 16 and 21 bp gapped SPS sites. Moreover, the recovery of DNA in the ChIP assay was also independent of transcriptionally inactivating mutations in the ankyrin domains. These results indicate that heterodimer complexes do exist on DNA, but since the recovery of dimer complexes was independent of both gap length and ankyrin domain function, this ChIP analysis was unable to determine if heterodimer NICD pairs are transcriptionally active.

### Head-to-head heterodimerization of NICD complexes affect transcriptional activation

**Figure 5:**
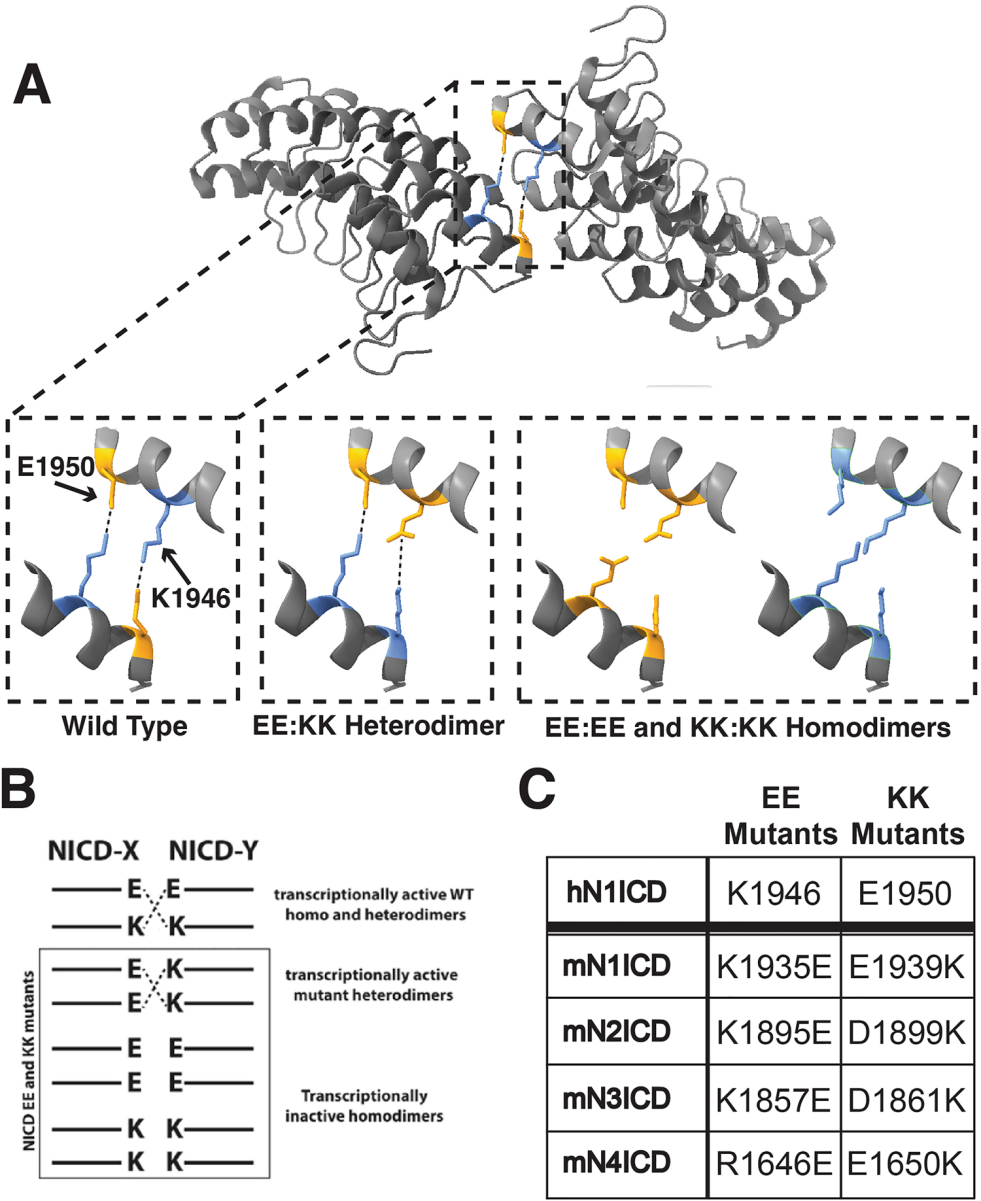
Homodimer blocking NICD compensatory mutation strategy. (**A**) Crystal structure of dimerizing human N1ICD ankyrin domains from Arnett et al.^13^. Positions of K1946 (blue) and E1950 (orange) are highlighted. Insets highlight charge interactions between adjacent NICDs and predicted interactions of NICD compensatory mutations. (**B**) Schematic representation of NICD compensatory mutations and predicted interactions. (**C**) Summary of amino acids mutated in mouse NICD molecules compared to human N1ICD.

Thus far, our results demonstrated that NICDs can engage in heterodimeric complexes in both the non-transcriptionally active yin-yang conformation as well as in the transcriptionally active head-to-head dimer conformation. However, none of our experiments had been able to determine if the head-to-head heterodimer complexes that form on DNA were in fact transcriptionally active. Assessing the transcriptional activity of heterodimer complexes is somewhat challenging since a simple reporter assay cannot distinguish between the transcriptional activities of homodimer and/or heterodimer complexes. Thus, we required a system where we would be able to directly monitor the transcriptional activity of heterodimers while excluding the transcriptional activity of homodimers. Drawing inspiration from Liu et al.^15^, and Nam et al.^12^, we created a series of compensatory NICD mutants that were incapable of homodimerization but still capable of heterodimerization. This strategy is based on the fact that NICD dimerization is mediated in part by salt bridges between positively charged lysine (K) residues and negatively charged glutamic acid (E) residues that interact across the interface of adjoining ankyrin domains as schematically illustrated in Fig. 5A and B. Nam et al., previously demonstrated that K1946E (EE) and E1950K (KK) mutation of human N1ICD thwarted homodimerization of EE or KK species but permitted homodimerization between N1ICD EE and KK species through restored salt bridge interactions across the interface between adjacent ankyrin domains. We hypothesized that a similar approach would allow us to monitor heterodimer formation between different species of mouse NICD molecules. To accomplish this, we first aligned the human N1ICD ankyrin domain with the ankyrin domains of the mouse Notch proteins to find the corresponding residues (Supplementary Fig. 1). Site directed mutagenesis was then used to mutate the mouse NICD molecules to contain lysine only (KK) or glutamic acid only (EE) residues at these dimerization sites as summarized in Fig. 5C.

**Figure 6:**
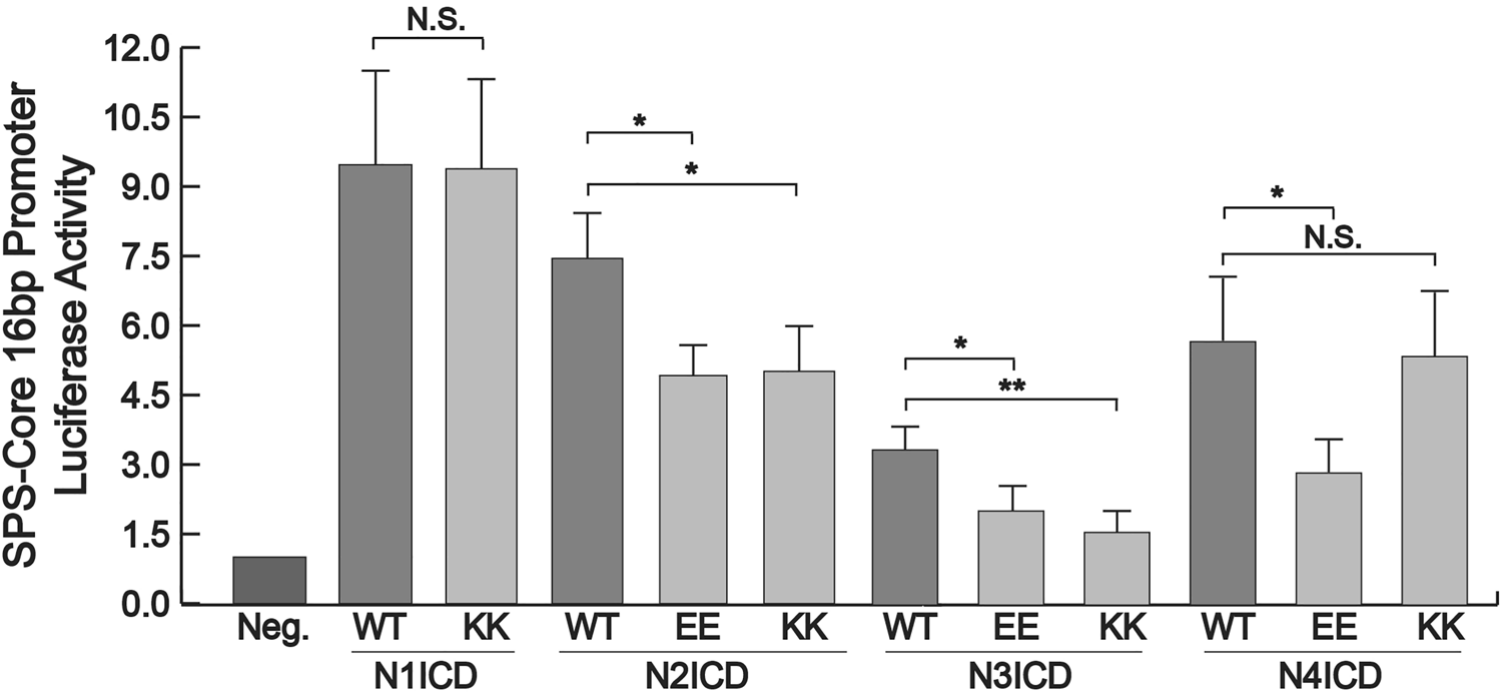
Luciferase assay activity of NICD dimer mutations. 293T cells were co-transfected with a SPS-Core 16 bp promoter luciferase reporter and wild-type (WT) or various NICD mutants. Reporter activation is compared to its basal activity in cells. Shown are the average +/− SE of three experiments. Statistical significance was determined through a student’s two-tailed t test, assuming equal variances, where *** is p < 0.001, ** is p < 0.01, and * is p < 0.05.

To test these mutants, we co-transfected 293T cells with various combinations of EE and KK mutants and luciferase reporters that have 16 bp gaps between their RBPj binding sites. The 16 bp gap has been shown to be the optimal distance between dimerizing NICDs to enable synergistic and robust transcriptional activity^17^. As shown in Fig. 6, the single N1ICD KK mutant we managed to generate did not significantly reduce activity compared to the wildtype N1ICD and for unknown reasons, we were never able to generate the second EE mutant in N1ICD. Despite this, the EE and KK mutants of both N2ICD and N3ICD significantly reduced luciferase activity. Finally, the EE but not KK N4ICD mutant, also considerably diminished reporter activity compared to control N4ICD.

**Figure 7:**
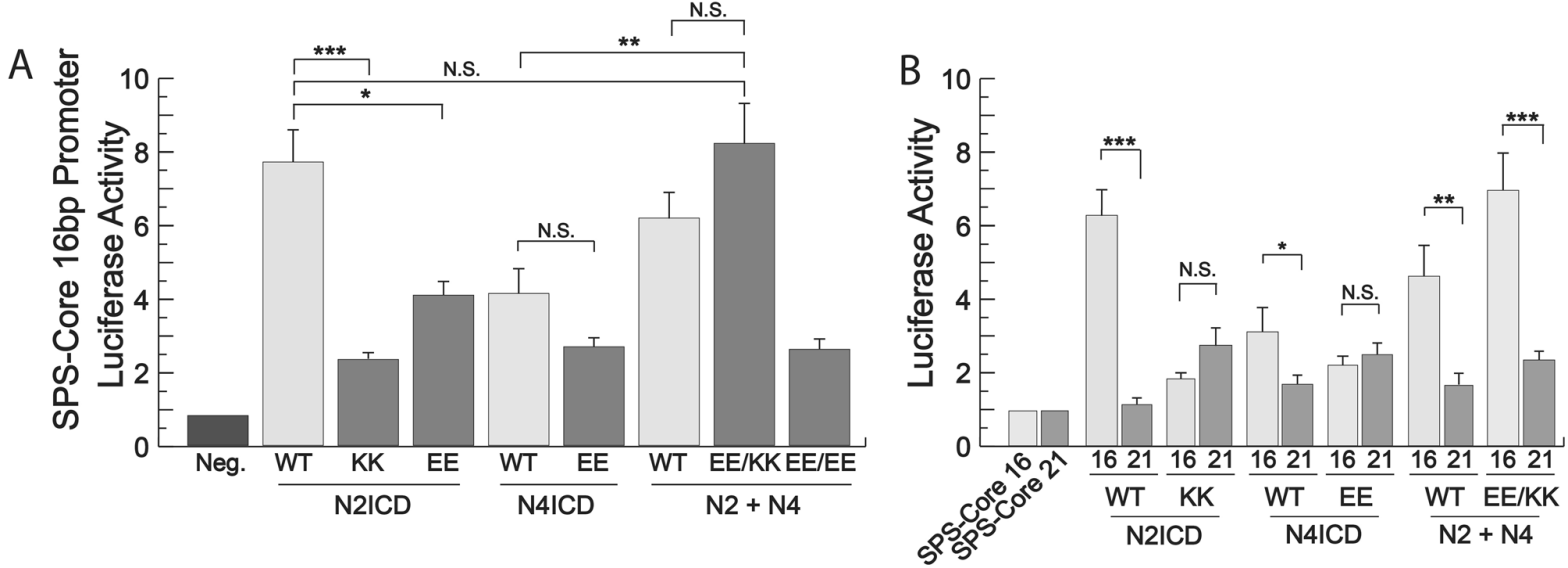
NICD heterodimers are transcriptionally active. (**A**) 293T cells were co-transfected with a SPS-Core 16 bp promoter luciferase reporter and either WT NICDs or N2ICD-KK, N2ICD-EE, N4ICD-EE or a combination of EE and KK mutants. Shown are the average +/− SE of six experiments. (**B**) Side-by-side comparison of results from 16 and 21 base pair promoters. Statistical significance was determined through a student’s two-tailed t test, assuming equal variances, where *** is p < 0.001, ** is p < 0.01, and * is p < 0.05.

Having found NICD mutations that were unable to support NICD homodimerization, we next sought to determine if these same mutant NICDs would be able to rescue transcriptional activity when KK and EE versions were combined. To accomplish this, we again co-transfected 293 T cells with the 16 bp gap luciferase reporter and various EE and KK combinations of mutant NICDs. We found that some but not all mutant NICD combinations were able to support transcription. For example, N2ICD KK and N4ICD EE both have reduced transcriptional activity compared to their non-mutated NICD counterparts indicating their inability to form homodimers. Moreover, co-transfection of N2ICD EE and N4ICD EE was unable to rescue transcription thus validating the need for EE and KK pairs (Fig. 7A). Most importantly however, co-transfection of N2ICD KK and N4ICD EE supported transcriptional activity comparable to wild-type N2ICD homodimers alone. A similar result was also observed when N2ICD KK and N3ICD EE mutants were co-transfected (Supplementary Fig. 2). Importantly, The N2ICD and N4ICD pair was unable to support strong transcriptional activity on a promoter with 21 bp between its RBPj binding sites (Fig. 7B). The longer base pair gap between TP-1 elements does not support synergistic and robust transcriptional activity, therefore this result indicated that the NICDs were not working individually to activate robust transcription. Despite the success of the N2ICD KK and N4ICD EE dimer pair and the N2ICD KK and N3ICD EE dimer pair, other KK/EE combinations failed to rescue robust transcriptional activity (Supplementary Fig. 2). Nonetheless, this data strongly supports the conclusion that the both aforementioned NICD combinations were forming legitimate heterodimers that synergistically activated transcription in a manner similar to normal homodimer pairs.

### Notch dimerization affects promoter activity with varying specificity

**Figure 8:**
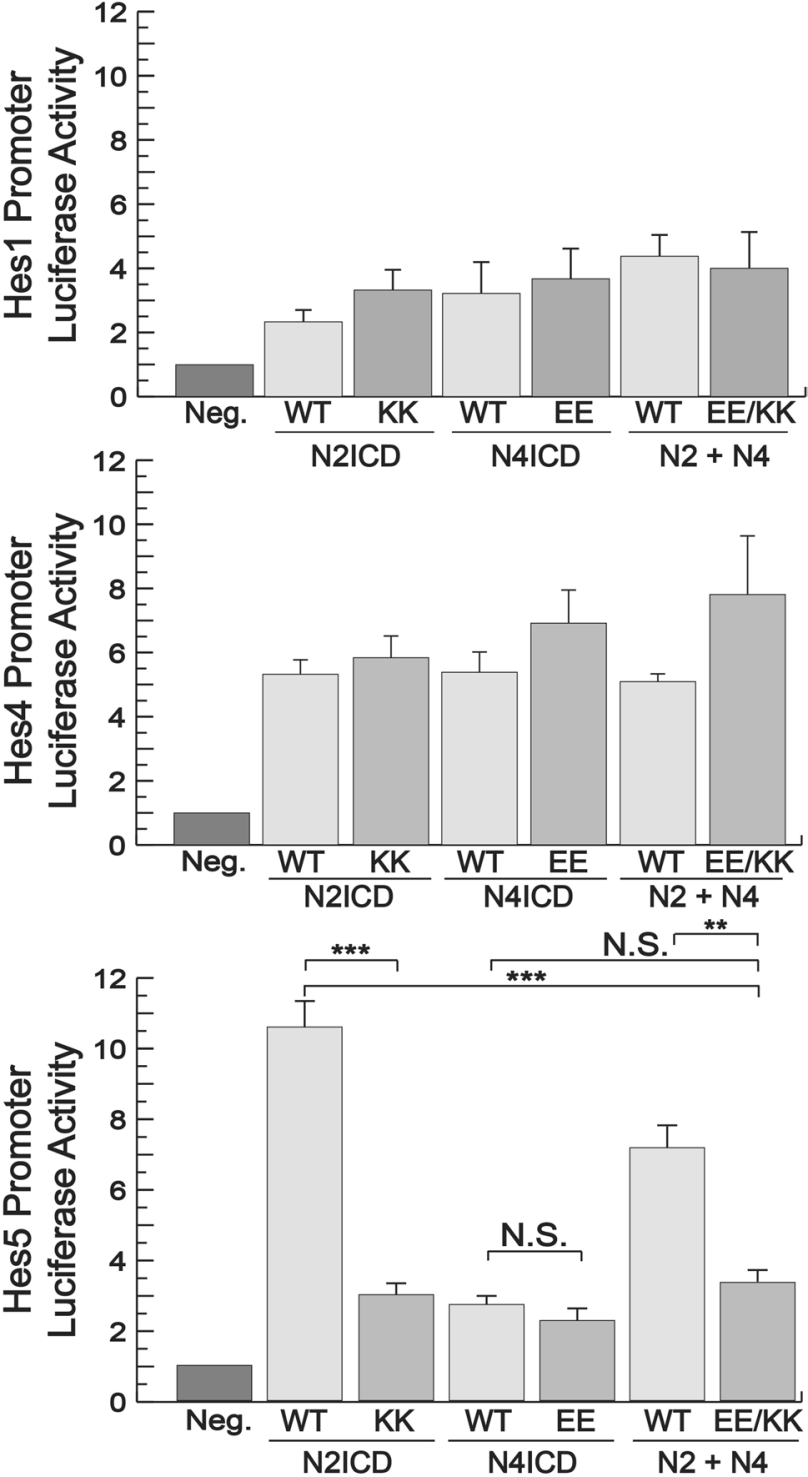
Differential activity of NICD heterodimers on various promoters. 293T cells were co-transfected with luciferase reporters containing either the Hes1 (top), Hes4 (middle), or Hes5 (bottom) promoters and either WT NICDs, N2ICD-KK, N4ICD-EE, or a combination of KK and EE NICDs. Hes1 data represents the average +/− SE of five experiments while Hes4 and Hes5 data represents the average +/− SE of four experiments. Statistical significance was determined through a student’s two-tailed t test, assuming equal variances, where *** is p < 0.001, ** is p < 0.01, and * is p < 0.05.

KK and EE compensatory mutations in the dimer domains of N2ICD and N4ICD rescued transcriptional activity using the optimized 16 bp SPS promoter suggesting heterodimerization between N2ICD-KK and N4ICD-EE. This promoter, however, is an artificially generated and optimized SPS element^17^ that does not contain flanking DNA sequences. Therefore, it was important to determine if these NICD pairs were also able to heterodimerize on more natural Notch-specific promoters. Using the same N2ICD-KK and N4ICD-EE mutants, we performed luciferase assays on the Hes1, Hes4, and Hes5 promoters that not only contain the SPS element, but also hundreds of base-pairs of flanking DNA sequences. Consistent with previous results^17^, the Hes1 and Hes4 promoters were only weakly activated by N2ICD or N4ICD transfection and interestingly, the EE and KK mutants had transcriptional activities comparable to their wild-type counterparts suggesting that these mutations failed to block homodimerization on these promoters. On the other hand, the N2ICD-KK but not the N4ICD-EE mutant was unable to form homodimers on the Hes5 promoter (Fig. 8). Surprisingly, the combination of the N2ICD-KK and N4ICD-EE mutants that rescued transcriptional activity of the synthetic promoter failed to rescue transcriptional activity on the Hes5 promoter.

## Discussion

Cell surface Notch receptors are activated by a well-documented series of proteolytic events that release transcriptionally active Notch intracellular domains from the cell membrane^4^. Upon release, NICD molecules travel to the nucleus where they assemble into complexes to drive the expression of Notch responsive genes^7,8^. In mammals, there are four Notch proteins that share significant structural homologies. In particular, all mammalian Notch proteins contain an ankyrin domain that is thought to mediate head-to-head dimerization between NICDs and thus allows dimerized NICDs to synergistically activate transcription from Notch promoters containing SPS elements^12,13^. This ankyrin mediated transcriptional activity has been found to be crucial for many of the activities attributed to Notch^3,15,23,24^.

The high degree of similarity between these ankyrin domains prompted us to hypothesize that the four mammalian NICDs might be able to engage in heterodimer formation. A requirement for this hypothesis is the expression of multiple Notch proteins in a single cell. We confirmed this by detection of at least two different mRNAs encoding Notch proteins in each cell line assayed. While we did not show that these mRNAs are translated, this result “set the stage” for NICD heterodimerization. We initially tested this heterodimerization hypothesis by performing co-immunoprecipitation experiments and discovered that all NICDs do seem to interact with each other and that N4ICD appeared to engage in these interactions stronger than the others. It should be noted, that transient overexpression of NICDs was required to observe co-immunoprecipitation thereby raising the possibility that these interactions may be dependent on the elevated protein concentrations associated with transient transfection. Moreover, since we were unable to precisely normalize protein expression levels, the relative strength of interactions will need to be validated by more precise measurements. Despite these limitations, we did determine that this interaction was not dependent on the ankyrin domains that are critical for the transcriptionally active head-to-head interaction of NICD subunits. Because of this, we believe our Co-IP approach did not detect head-to-head NICD dimers that depend on ankyrin interactions but instead detected the anti-parallel “yin-yang” conformation previously described by Vasquez et al.^16^.

Vasquez et al., concluded that the function of this yin-yang complex is to help assemble the complete NICD transcriptional complex by loading MAML onto NICD but that this complex is not in itself transcriptionally active. Since the goal of our current investigation was to determine if NICD molecules could assemble into heterodimeric head-to-head transcriptional complexes, we chose not to pursue this avenue of research at this time. Nonetheless, our results expanded the understanding of this yin-yang complex. In particular, if the function of the yin-yang complex is to load MAML onto NICD as described previously, our results suggest that N4ICD may perform this task more effectively than the other NICDs. If this is the case, we would predict that stronger yin-yang interactions should correlate to enhanced transcriptional activity which is not what we observed. Instead, we and others have found that N1ICD and N2ICDs have considerably stronger transcriptional activities compared to N4ICD even though N4ICD appears to outcompete the other NICDs in forming yin-yang dimers. Likewise, N3ICD is transcriptionally weak, but also inefficient at forming yin-yang dimers compared to N4ICD. One possibility to explain these results is the absence of a transcriptional activation domain (TAD) and a short C-terminal in N4ICD suggesting that these elements may act to reduce yin-yang dimerization. In the end however, the connection between the yin-yang complex and transcriptional activity does not appear to be straight forward and will require additional investigation to understand the function of this understudied NICD complex.

Interestingly, we noticed that the N1ICD homodimers appeared to form the weakest yin-yang dimers, with inconsistent Co-IP results. To remedy this, we employed a C-terminally truncated N1ICD construct which effectively rescued the unreliable precipitation of the homodimers. This led us to hypothesize that there may be an inhibitory domain on the C-terminal of the N1ICD that reduces yin-yang complex formation. A tempting explanation for this arises from a recent pre-print that describes what appear to be membraneless nuclear condensates containing Notch^25^. In this pre-print, the authors find that Notch1 aggregation into these nuclear condensates appears to be dependent on an intrinsically disorganized domain (IDR) in the C-terminus of N1ICD. Although this data is still unpublished and preliminary, it is consistent with observations from other groups showing that N1ICD does aggregate in nuclear puncta even when expressed at endogenous levels^26–29^. It is therefore tempting to speculate that yin-yang dimerization is somehow related to the formation of these Notch containing nuclear puncta. This would be consistent with our observation that the N1ICD C-terminal truncation (which may have deleted or truncated the IDR) performed better in Co-IP experiments, although this will need be more closely investigated.

We next attempted to detect head-to-head NICD heterodimers with ChIP and found both N1ICD and N4ICD binding to a transcriptionally active Notch responsive promoter with a 16 bp gap between the RBPj binding sites^17^. Since 16 base-pairs is the optimal distance between RBPj binding sites to support transcription, it is very likely that this N1ICD-N4ICD pair was a heterodimer transcription complex. However, we also found that this apparent heterodimer pair bound to transcriptionally weak Notch promoters with 21 base-pairs between RBPj binding sites^17^ with apparently similar affinity. So, although we demonstrated heterodimer formation by ChIP, it was not clear whether these heterodimers were transcriptionally active or were individually binding to RBPj irregardless of transcriptional potential.

To address this issue of determining if heterodimer pairs are transcriptionally active, we turned to a system of compensatory mutations to specifically block NICD homodimer formation but allow heterodimer formation. This approach was necessary because any two NICD pairs expressed in a cell could potentially drive transcription through a combination of homodimer and/or heterodimer pairs, but transcription reporter (luciferase) assays cannot distinguish between these types of dimerization, just overall promoter activity. Using this approach, our data suggests that at least two combinations of NICD heterodimers (N2ICD-N4ICD and N2ICD-N3ICD) are able to form transcriptionally active complexes. Using a similar technique, Kobia et al.^23^ previously observed N1ICD-N2ICD heterodimers suggesting that additional heterodimer pairs may be possible. Given that N4ICD is transcriptionally less active than N2ICD, we originally predicted that N2ICD-N4ICD heterodimers would have transcriptional activities somewhere in between N2ICD and N4ICD homodimers. However, we found that N2ICD-N4ICD heterodimers demonstrated transcriptional activity on par with N2ICD homodimers when tested on the synthetic SPS-Core reporter construct. This construct features a minimal Notch responsive element consisting of only two optimized TP1 RBPj binding sites separated by 16 base pairs^17^. In comparison, when this same heterodimer pair was tested on the natural Hes1 and Hes4 promoters, there was no apparent difference between homodimer and heterodimer combinations. This failure may be due to the presence of other non-SPS binding sites for RBPj which function independently of NICD dimerization thus masking the contribution of heterodimerized NICDs. On the contrary, N2ICD-N4ICD heterodimers demonstrated transcriptional activity more similar to N4ICD on the natural Hes5 promoter than the N2ICD homodimer. In comparison, the N1ICD-N2ICD heterodimer pair described by Kobia et al.^23^ was shown to have greater transcriptional activity than either N1ICD or N2ICD homodimers. The reason for this promoter specificity is not known, but taken at face value, these results suggest that NICD heterodimers can assemble on natural promoters and that heterodimer complexes have transcriptional activities distinct from their corresponding homodimers. Future experiments with chromosomally located promoters as well as with full-length Notch proteins that are activated via normal signaling should be performed to help clarify these observations.

The implications of this finding will need to be explored; however, several possibilities present themselves. First, it is well documented that the four mammalian NICDs have varying transcriptional activities. Given this, NICD heterodimers might have different transcriptional strengths compared to homodimers which is supported by our data showing that the N2ICD- N4ICD heterodimer had transcriptional activity more similar to N4ICD than to N2ICD on the Hes5 promoter. Since many cell lines express more than one Notch receptor, such an activity might increase the importance of the otherwise transcriptionally weak N3ICD and N4ICD molecules since these molecules might serve to modulate the more robust transcriptional activities of N1ICD and N2ICD. In addition, the various NICDs are subject to a range of post-transcriptional modifications which when combined with heterodimerization may present yet more possibilities to diversify Notch signaling.

In conclusion, the results presented here expand our basic understanding of the Notch signaling pathway. Major outstanding questions that remain include the formation and function of the yin-yang NICD dimerization complex(s), the extent to which NICD heterodimerization influences Notch signaling output, and if Notch homodimerization or heterodimerization can be regulated by cell signaling. Answering these questions will shed light on Notch signaling and help to understand how this ubiquitous signaling mechanism regulates cell biology.

### Supplementary Information

Supplementary Tables.

Supplementary Figure 1.

Supplementary Figure 2.

Supplementary Legends.
