# Supplementary material for: Notch intracellular domains form transcriptionally active heterodimeric complexes on sequence-paired sites
Source: Sci Rep. 2024 Jan 2;14:218. doi: 10.1038/s41598-023-50763-4 (PMC10761890; doi:10.1038/s41598-023-50763-4)
Supplement: Supplementary file 1 — Supplementary Tables. [file 41598_2023_50763_MOESM1_ESM.docx]

**Supplemental Tables**

**Supplementary table 1: Primers used for RT-PCR Analysis of the Notch Expression Profiles.**

| Notch1 | FWD | AGGTCAATGAGTGCAACAGCAACC |
| --- | --- | --- |
|  | REV | TCAATACACGTGCCCTGGTTCAGA |
| Notch2 | FWD | AATGGTGGCACATGTGTTGATGGG |
|  | REV | ACATAGGCACTGGGACTCTGCTTT |
| Notch3 | FWD | GATGGCATGGATGTCAATGTGCGT |
|  | REV | AGCGGTTTCGGATGAGAATCTGGA |
| Notch4 | FWD | ACTCCTGTGCCACTTGGAAGACAT |
|  | REV | AGAGGCACTCATTGTGATCAGCCT |
| 18S | FWD | CAGCCACCCGAGATTGAGCA |
|  | REV | TAGTAGCGACGGGCGGTGTG |

**Supplementary Table 2: Primers used for Chromatin Immunoprecipitation Analysis**

| Human Hes1 Promoter Primer for Notch-Dimer Driven ChIP | FWD | TCCTCCCATTGGCTGAAA |
| --- | --- | --- |
|  | REV | GGCCTCTATATATATCTGGGACTG |
| Human Hes4 Promoter Primer for Notch-Dimer Driven ChIP | FWD | CGAGGCGTGACTGACA |
|  | REV | CAGGCCGTTTCCCTATTTAAG |

**Supplementary Table 3: Constructs cloned with restriction enzymes.**

| **Plasmid**  **Number** | **Desired Construct/ Primer Function** | **Dir.** | **Sequence (5'→3')** |
| --- | --- | --- | --- |
| 285  N1ICD-MYC | Transfer N1ICD into pcDNA3.1-MycHis B | FWD (KPN1) | TTATTAGGTACCACCATGGTGCTGCTGTCCCGC |
|  |  | REV (XBA1) | TTATTATCTAGAGCTTTAAATGCCTCTGGAATGTGG |
| 286  N2ICD-MYC | Transfer N2ICD into pcDNA3.1-MycHis B | FWD (KPN1) | GGCGGCGGTACCACCATGATGGCCAAGCGGAAGCG |
|  |  | REV (XBA1) | GGCGGCTCTAGATGCATACACCTGCATGTTGCTGTG |
| 287  N3ICD-MYC | Transfer N3ICD into pcDNA3.1-MycHis B | FWD (KPN1) | GGCGGCGGTACCACCATGATGGTTGCCAGGCGAAGC |
|  |  | REV (XBA1) | GGCGGCTCTAGAGGCCATCACCTGCCTCTTG |
| 288  N4ICD-MYC | Transfer N4ICD into pcDNA3.1-MycHis B | FWD (KPN1) | GGCGGCGGTACCACCATGGTCCTCCAGCTCATTCGG |
|  |  | REV (XBA1) | GGCGGCTCTAGAGCGTTCAGATTTCTTACAACCGAGTTTAAGGG |
| 296  N1ICD-HA | Transfer N1ICD into pKH3 | FWD (SAL1) | TTATTAGTCGACACCATGGTGCTGCTGTCCCGC |
|  |  | REV (XBA1) | TTATTATCTAGATTTAAATGCCTCTGGAATGTGG |
| 297  N2ICD-HA | Transfer N2ICD into pKH3 | FWD (SAL1) | GGCGGCGTCGACACCATGATGGCCAAGCGGAAGCG |
|  |  | REV (XBA1) | GGCGGCTCTAGATGCATACACCTGCATGTTGCTGTG |
| 298  N3ICD-HA | Transfer N3ICD into pKH3 | FWD (SAL1) | GGCGGCGTCGACACCATGATGGTTGCCAGGCGAAGC |
|  |  | REV (XBA1) | GGCGGCTCTAGAGGCCATCACCTGCCTCTTG |
| 299  N4ICD-HA | Transfer N4ICD into pKH3 | FWD (SAL1) | GGCGGCGTCGACACCATGGTCCTCCAGCTCATTCGG |
|  |  | REV (XBA1) | GGCGGCTCTAGAGTTCAGATTTCTTACAACCGAGTTTAAGGG |
| 391  Hes4-Luciferase | Clone Hes4 promoter to pGL3-Basic | FWD (KPN1) | GGCCGGGGTACCCGAGGCGTGACTGACA |
|  |  | REV (SAC1) | GGCCGGGAGCTCCAGGCCGTTTCCCTATTTAAG |

**Supplementary Table 4: Constructs cloned with partially overlapping primers:**

| **Plasmid #**  **Plasmid name** | **Desired Construct/ Primer Function** | **Dir.** | **Sequence (5'→3')** |
| --- | --- | --- | --- |
| 281  N1ICD-FLAG (R1974A) | Mutagenesis of N1ICD ankyrin domain R1974A | FWD | CCGGAACGCCGCCACAGATCTGGATGCCCGAATGC |
| 355  N1ICD-MYC (R1974A) |  |  |  |
| 289  N1ICD-MYC (R1974A) (ΔS2184) |  | REV | GTGGCGGCGTTCCGGAGCAGGATCTGGAAGACACC |
| 358  N1ICD-HA (R1974A) |  |  |  |
| 282  N2ICD-FLAG (R1934A) | Mutagenesis of N2ICD ankyrin R1934A | FWD | CCGCAACGCCGTAACCGATCTGGATGCCAGAATGA |
| 356  N2ICD-MYC (R1934A) |  |  |  |
| 359  N2ICD-HA (R1934A) |  | REV | GTTACGGCGTTGCGGATCAGAATCTGAAAGACACCTTGGGC |
| 283  N3ICD-FLAG (R1896A) | Mutagenesis of N3ICD ankyrin R1896A | FWD | CAGGAACGCCTCCACTGACCTGGATGCCCGAATGG |
| 357  N3ICD-Myc (R1896A) |  |  |  |
| 360  N3ICD-HA (R1896A) |  | REV | GTGGAGGCGTTCCTGATGAGAATCTGGAAGACACCCTGGG |
| 284  N4ICD-Flag (R1685A) | Mutagenesis of N4ICD ankyrin R1685A | FWD | CCAGCGCCCAGACTACGGTGGACG |
| 290  N4ICD-Myc (R1896A) |  | REV | GTCTGGGCGCTGGCCAATAGGAGCT |
| 361  N4ICD-HA (R1896A) |  |  |  |

**Supplementary Table 5: Constructs cloned with non-overlapping primers.**

| Notch1 E1939K (Flag) | FWD | AAGGCCAGTGCAGATGCCAAC |
| --- | --- | --- |
|  | REV | CAGCAAGCGCTTTGCAGCATC |
| Notch2 K1895E (Flag & Myc) | FWD | GAGCGCCTCCTGGATGCT |
|  | REV | GGCAGCATCAGCTCTCGA |
| Notch2 D1861K (Flag & Myc) | FWD | AAAGCTGGTGCGGATGCAAAT |
|  | REV | CAGGAGGCGTTTGGCAGCATC |
| Notch3 K1857E (Flag) | FWD | GAGCGTCTCCTGGATGCTGGG |
|  | REV | GGCTGCATCCGCTCTAGCATA |
| Notch3 D1861K | FWD | CTCCTGAAGGCTGGGGCGGACAC |
|  | REV | CCCAGCCTTCAGGAGACGCTTGGC |
| Notch4 R1646E | FWD | GAGCGCCTCCTTGAGGCTGGAGCC |
|  | REV | GGCAGCGGTTGGCCGAGA |
| Notch4 E1650K | FWD | AAGGCTGGAGCCAACCCCAAC |
|  | REV | AAGGAGGCGGCGGGC |
