# Supplementary figures and images for: Notch intracellular domains form transcriptionally active heterodimeric complexes on sequence-paired sites
Source: Sci Rep. 2024 Jan 2;14:218. doi: 10.1038/s41598-023-50763-4 (PMC10761890; doi:10.1038/s41598-023-50763-4)

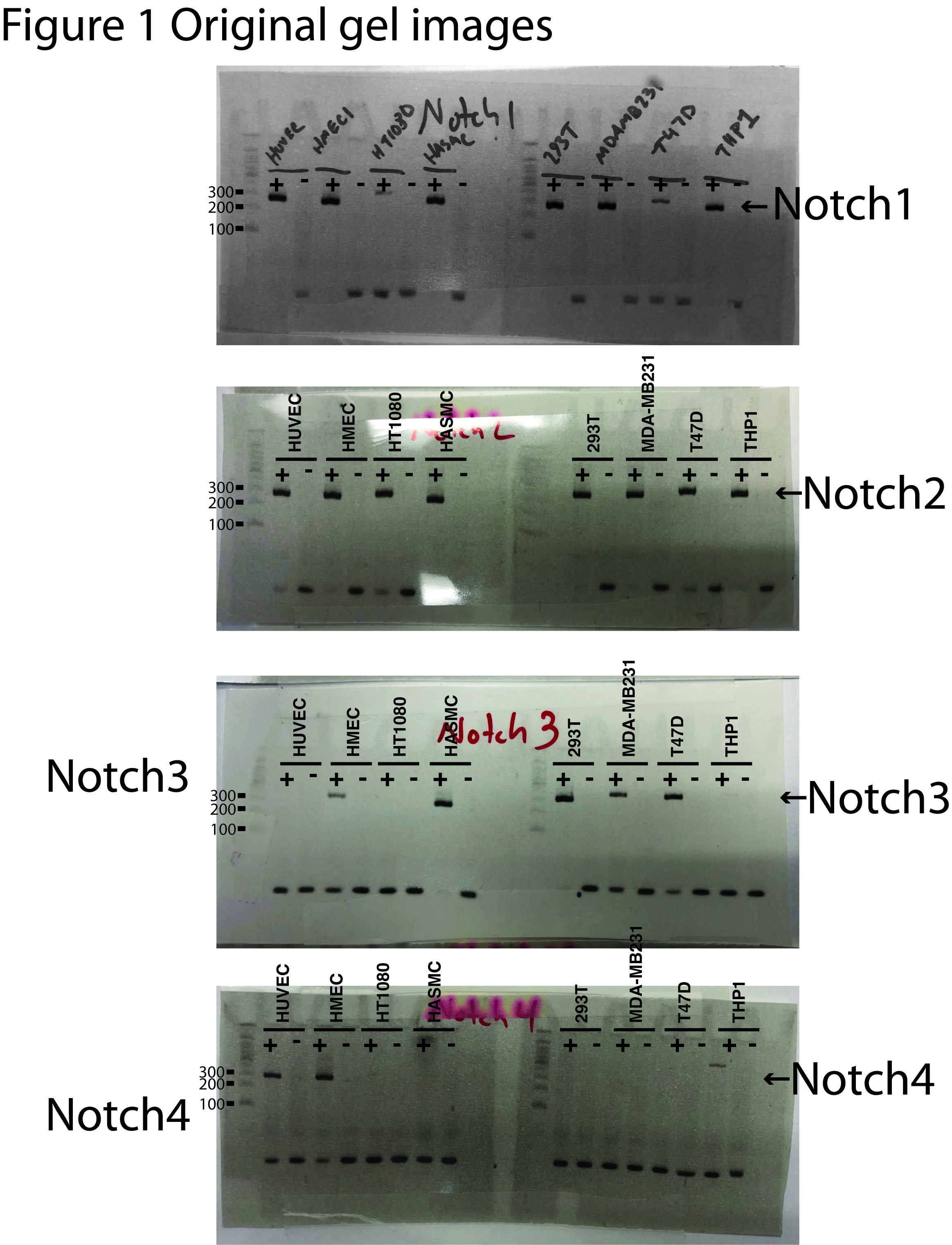

Supplement: Supplementary file 2 — Supplementary Figure 1. [file 41598_2023_50763_MOESM2_ESM.tif]

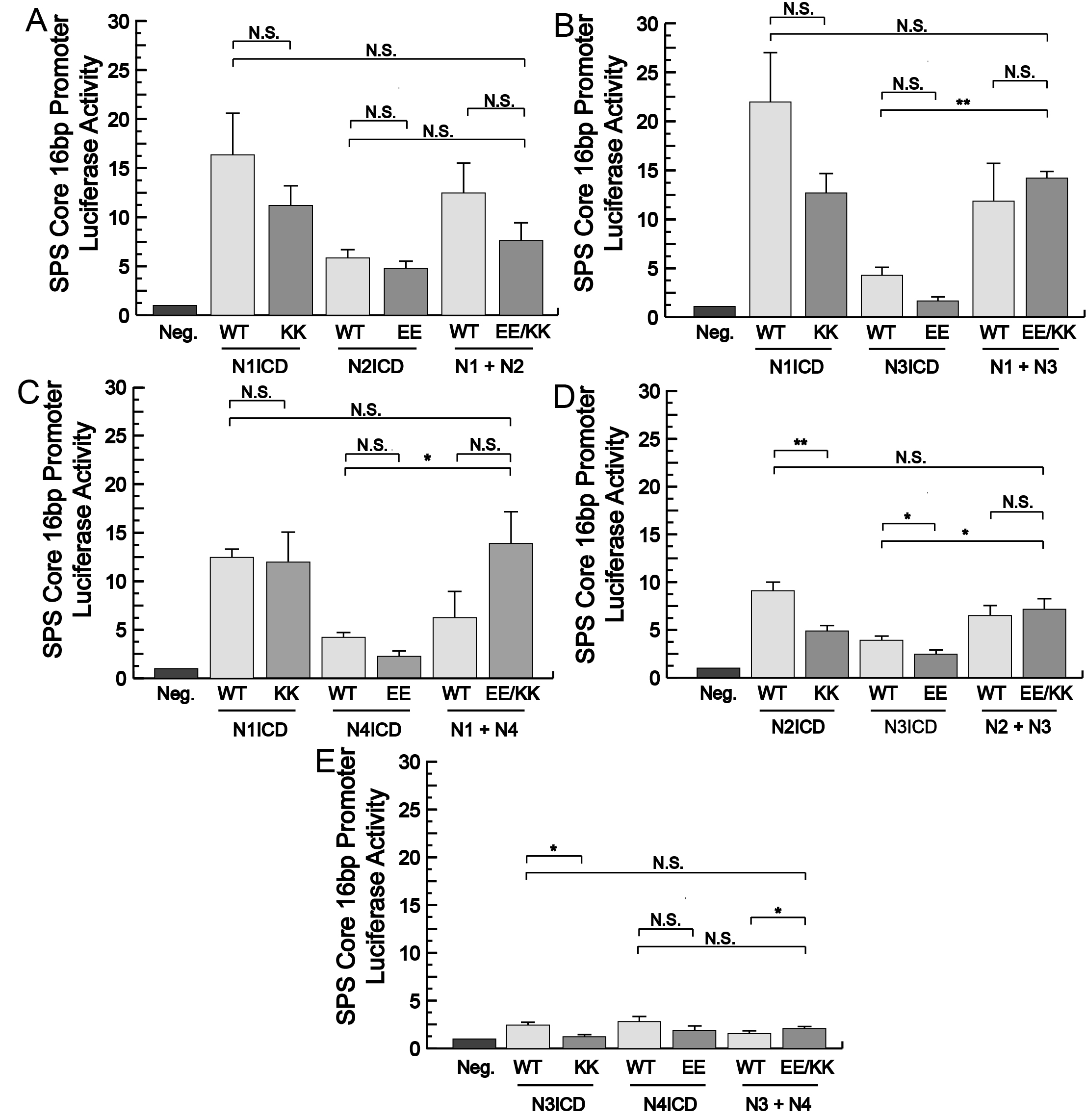

Supplement: Supplementary file 3 — Supplementary Figure 2. [file 41598_2023_50763_MOESM3_ESM.jpg]
