# Supplementary material for: Notch intracellular domains form transcriptionally active heterodimeric complexes on sequence-paired sites
Source: Sci Rep. 2024 Jan 2;14:218. doi: 10.1038/s41598-023-50763-4 (PMC10761890; doi:10.1038/s41598-023-50763-4)
Supplement: Supplementary file 4 — Supplementary Legends. [file 41598_2023_50763_MOESM4_ESM.docx]

**Supplementary Figure 1. Alignment of mouse Notch dimerization sites to known human Notch sites.** Mouse Notch1-4 amino acid sequences from NCBI were aligned to the human Notch1 sequence. Previously discovered Notch interaction sites are shown at amino acids K1946, E1950, R1983, and R1985 on human Notch.

**Supplementary Figure 2. Testing of Notch H-H dimerization using different NICD mutants.**

293T cells were co-transfected with a promoter luciferase reporter and wild-type (WT) or corresponding binding site mutant (Mut) NICDs. Reporter activation is compared to its basal activity in cells. Transfection efficiency was normalized by co-transfection with CMV-β-gal reporter plasmids and measuring β-gal activity. (**A**) Cells were co-transfected with a SPS-Core 16bp promoter luciferase reporter and either WT or Mutant N1ICD, N2ICD or a combination of the two (N1 E1939K, N2 K1895E). Shown are the average +/- SE of seven experiments. (**B**) Cells were co-transfected with a SPS-Core 16bp promoter luciferase reporter and either WT or Mut N1ICD, N3ICD or a combination of the two (N1 E1939K, N3 K1857E). Shown are the average +/- SE of three experiments. (**C**) Cells were co-transfected with a SPS-Core 16bp promoter luciferase reporter and either WT or Mut N1ICD, N4ICD or a combination of the two (N1 E1939K, N4 R1646E). Shown are the average +/- SE of three experiments. (**D**) Cells were co-transfected with a SPS-Core 16bp promoter luciferase reporter and either WT or Mut N2ICD, N3ICD or a combination of the two (N2 D1861K, N3 K1857E). Shown are the average +/- SE of six experiments. (**E**) Cells were co-transfected with a SPS-Core 16bp promoter luciferase reporter and either WT or Mut N3ICD, N4ICD or a combination of the two ( N3 D1861K, N4 R1646E). Shown are the average +/- SE of five experiments. Statistical significance was determined through a student’s two-tailed *t* test, assuming equal variances, where *** is p < 0.001, ** is p < 0.01, and * is p < 0.05.
